# A Mathematical Study of a TB Model with Treatment Interruptions and Two Latent Periods

**DOI:** 10.1155/2014/932186

**Published:** 2014-05-22

**Authors:** Luju Liu, Yan Wang

**Affiliations:** ^1^School of Mathematics and Statistics, Henan University of Science and Technology, Luoyang 471023, China; ^2^College of Science, China University of Petroleum, Qingdao, Shandong 266580, China

## Abstract

A TB transmission model which incorporates treatment interruptions and two latent periods is presented. The threshold parameter known as the control reproduction number and the equilibria for the model are determined, and the global asymptotical stabilities of the equilibria are studied by constructing the proper Lyapunov functions. The reproduction numbers and numerical simulations show that treatment of active TB cases always helps to control the TB epidemic, while treatment interruptions may have a negative, positive, or no effect on combating TB epidemic.

## 1. Introduction

Tuberculosis (TB) caused by infection with the bacillus* Mycobacterium tuberculosis* (*M. tuberculosis*) is a very common and an infectious airborne disease. It typically affects the lungs (pulmonary TB) but can affect other sites as well (extrapulmonary TB). It is estimated that one-third of the world's population has been infected with the* M. tuberculosis* [1]. Moreover, an estimated 8.6 million people developed TB and 1.3 millon died from the disease (including 320 thousand deaths among HIV-positive people) in 2012 [2]. Although the rate of new TB cases and the TB incidence rates are falling worldwide and the TB mortality rate has been reduced, the absolute number of incident cases of TB is increasing due to population growth [2, 3]. Therefore, TB remains a major global health problem [2].

In 2011, the treatment success rate continued to be high at 87% among all new TB cases [2]. However, there were about 3 million people who developed TB and were missed by national notification systems [2]. On the other hand, treatment interruptions are frequent in active TB cases during the intensive phase and the continuation phase because of a wide range of reasons [4]. It may be recognized that treatment interruptions and the missed TB cases are the key factors to cause the more drug-resistant TB cases and the high TB mortality [4, 5]. The factor of treatment interruptions may result in more susceptible people infected as well. In 2012, there was an estimation that 450 thousand individuals developed multidrug-resistant TB (MDR-TB) and an estimation of 170 thousand deaths from MDR-TB [2], which is currently a main threat to tuberculosis control programs and community health [6].

When susceptible people are infected, they enter a latent stage which varies from person to person. Most of them carry the bacillus* M. tuberculosis* for 20 or 30 years and do not progress active TB cases. In [7], Ziv et al. first considered two latency periods. In [8–11], the period of latency has been introduced into the mathematical models associated with TB. In particular, [10] developed a TB model with two parallel latency periods and different progressions in order to study the globally asymptotical stability of the endemic equilibrium. In [11], a multidrug- resistant TB model incorporating exogenous reinfection, two latency periods, and two treatment stages of active TB cases was formulated to examine the stabilities of the equilibria. Therefore, the latency period of tuberculosis can not be neglected because of its importance in analyzing the TB models. In the present paper, we pay our attention to the factors of treatment interruptions and two latent periods.

The present paper is built up as follows. In Section 2, we outline the mathematical TB model. We study the stabilities of equilibria of the model system in Section 3. In Section 4, the effects of treatment of active TB cases and treatment interruptions on the development of TB epidemic are considered. The paper ends with a brief conclusion.

## 2. The Mathematical Model

**Figure 1:**
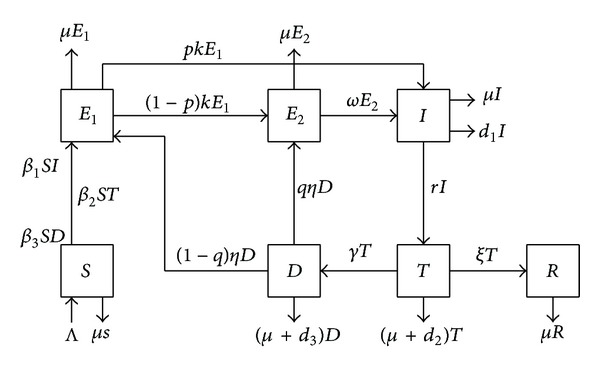
The transfer diagram of the TB transmission model.

In this section, the TB transmission model is formulated. The whole population is divided into seven groups according to their epidemiological status. The groups are the susceptible people (*S*(*t*)), the early latent people (*E*_1_(*t*)), the later latent people (*E*_2_(*t*)), the untreated active TB cases (who have not been treated yet) (*I*(*t*)), the treated active TB cases (who have been treated) (*T*(*t*)), the active TB cases who have interrupted treatment (*D*(*t*)), and the removed people (*R*(*t*)), respectively, where *t* is the time variable. It is assumed that once the treatment of active TB cases is interrupted, there is no more treatment. The mass action incidence is used here. The transmission diagram is given in Figure 1, and the mathematical model is described by the following system of ordinary differential equations:

(1) $$\begin{array}{c} \frac{dS}{dt}=\Lambda -\beta_{1}SI-\beta_{2}ST-\beta_{3}SD-\mu S,\\ \frac{dE_{1}}{dt}=\beta_{1}SI+\beta_{2}ST+\beta_{3}SD+\left(1-q\right)\eta D-\left(\mu +k\right)E_{1},\\ \frac{dE_{2}}{dt}=\left(1-p\right)kE_{1}+q\eta D-\left(\mu +\omega \right)E_{2},\\ \frac{dI}{dt}=pkE_{1}+\omega E_{2}-\left(\mu +d_{1}+r\right)I,\\ \frac{dT}{dt}=rI-\left(\mu +d_{2}+\gamma +\xi \right)T,\\ \frac{dD}{dt}=\gamma T-\left(\mu +d_{3}+\eta \right)D,\\ \frac{dR}{dt}=\xi T-\mu R. \end{array}$$

In system (1), Λ stands for the recruitment rate of the susceptible population. *μ* is the percapital natural death rate. *d*_*i*_  (*i* = 1,2, 3) is the disease induced death rate in classes *I*, *T*, and *D*, respectively. It is natural to assume that *d*_1_ > *d*_3_ > *d*_2_ due to the treatment of active TB cases reducing the disease induced death rate. *β*_*i*_  (*i* = 1,2, 3) is the transmission coefficients from class *I*, class *T*, and class *D* to class *S*, respectively. We assume that *β*_1_ > *β*_3_ > *β*_2_ because the treatment of active TB cases reduces the infectivity of active TB cases. Take *p*  (0 ≤ *p* ≤ 1) as the fraction of the early latent persons who have fast TB progression. *k* is the reactivation rate of the early latent persons. *ω* is the reactivation rate of the later long-term latent persons. *η* is the self-cured rate of the persons in class *D* because of their immune system being strengthened. *q*  (0 ≤ *q* ≤ 1) is the fraction of the self-cured persons in class *D* who enter class *E*_2_. *r* is the treatment rate of the untreated active TB cases. *γ* is the rate of treatment interruptions in class *T*. *ξ* stands for the recovery rate of the treated active TB cases. It is assumed that all the parameters are nonnegative based on the biological consideration.

Since *R* does not appear in the first six equations of system (1), it is necessary to discuss the following equivalent system instead of system (1):

(2) $$\begin{array}{c} \frac{dS}{dt}=\Lambda -\beta_{1}SI-\beta_{2}ST-\beta_{3}SD-\mu S,\\ \frac{dE_{1}}{dt}=\beta_{1}SI+\beta_{2}ST+\beta_{3}SD+\left(1-q\right)\eta D-\left(\mu +k\right)E_{1},\\ \frac{dE_{2}}{dt}=\left(1-p\right)kE_{1}+q\eta D-\left(\mu +\omega \right)E_{2},\\ \frac{dI}{dt}=pkE_{1}+\omega E_{2}-\left(\mu +d_{1}+r\right)I,\\ \frac{dT}{dt}=rI-\left(\mu +d_{2}+\gamma +\xi \right)T,\\ \frac{dD}{dt}=\gamma T-\left(\mu +d_{3}+\eta \right)D. \end{array}$$

It then follows from [12, Theorem  5.2.1] that, for any initial value in *R*_+_^6^, system (2) has a unique local nonnegative solution through the given initial value.

In the present paper, *N*(*t*) denotes the number of the total population in time *t*. That is,

(3) $$\begin{array}{c} N=S+E_{1}+E_{2}+I+T+D+R. \end{array}$$

Adding the equations in model (1) gives

(4) $$\begin{array}{c} \frac{dN}{dt}=\Lambda -\mu N-d_{1}I-d_{2}T-d_{3}D\leq \Lambda -\mu N. \end{array}$$

It is quite clear that if *N* > Λ/*μ* and *dN*/*dt* < 0. Therefore, all the solutions of system (2) with nonnegative initial values in the space *R*_+_^6^ are bounded and exist on the interval [0, +*∞*). Moreover, it is easily shown that the set

(5) $$\begin{array}{c} \Omega =\left\{\left(S,E_{1},E_{2},I,T,D\right)\in R_{+}^{6}\;|\;S\leq N\leq \frac{\Lambda }{\mu }\right\} \end{array}$$

is positively invariant and attracts all nonnegative solutions of model (2). Therefore, without loss of generality, it is only necessary to consider the solutions of model (2) with initial values in *Ω*.

To simplify the presentation, we let

(6) $$\begin{array}{c} A_{1}=\frac{\left(1-p\right)k}{\mu +k},\;\;A_{2}=\frac{pk}{\mu +k},\\ A_{3}=\frac{\omega }{\mu +\omega },\;\;A_{4}=\frac{r}{\mu +d_{1}+r},\\ A_{5}=\frac{\gamma }{\mu +d_{2}+\gamma +\xi },\;\;A_{6}=\frac{\left(1-q\right)\eta }{\mu +d_{3}+\eta },\\ A_{7}=\frac{q\eta }{\mu +d_{3}+\eta },\;\;A_{8}=\left(\mu +d_{1}+r\right)-A_{3}A_{5}A_{7}r,\\ A_{9}=\left(\mu +d_{1}+r\right)-A_{3}A_{5}A_{7}r-A_{1}A_{3}A_{5}A_{6}r-A_{2}A_{5}A_{6}r. \end{array}$$

Clearly, the system (2) possesses the disease-free equilibrium *P*_0_(Λ/*μ*, 0,0, 0,0, 0) for all the parameters. The control reproduction number can be calculated by using the next generation matrix method [13]. The matrices *F* and *V* for the new infection terms and the remaining transfer terms are given by

(7) $$\begin{array}{c} F=\left(\begin{array}{ccccc} 0 & 0 & \beta_{1}\frac{\Lambda }{\mu } & \beta_{2}\frac{\Lambda }{\mu } & \beta_{3}\frac{\Lambda }{\mu }\\ 0 & 0 & 0 & 0 & 0\\ 0 & 0 & 0 & 0 & 0\\ 0 & 0 & 0 & 0 & 0\\ 0 & 0 & 0 & 0 & 0 \end{array}\right),\\ V=\left(\begin{array}{ccccc} \mu +k & 0 & 0 & 0 & -\left(1-q\right)\eta \\ -\left(1-p\right)k & \mu +\omega & 0 & 0 & -q\eta \\ -pk & -\omega & \mu +d_{1}+r & 0 & 0\\ 0 & 0 & -r & \mu +d_{2}+\gamma +\xi & 0\\ 0 & 0 & 0 & -\gamma & \mu +d_{3}+\eta \end{array}\right), \end{array}$$

respectively. Therefore, the control reproduction number *R*_*ti*_ [13] can be computed as follows:

(8) Rti=ρ(FV−1)=β1Λμ(A2+A1A3)×((μ+d1+r)−A3A5A7r  −A1A3A5A6r−A2A5A6r)−1+β2Λμ(A2A4+A1A3A4)×((μ+d2+γ+ξ)−A3A4A7γ  −A1A3A4A6γ−A2A4A6γ)−1+β3Λμ(A2A4A5+A1A3A4A5)×((μ+d3+η)−A3A4A5qη  −A1A3A4A5(1−q)η−A2A4A5(1−q)η)−1,

where *ρ*(*M*) is the spectral radius of matrix *M*. The control reproduction number, *R*_*ti*_, is interpreted as follows:

1. *A*_1_ = (1 − *p*)*k*/(*μ* + *k*) is the fraction that survives the early latent period and enters the later latent period;
2. *A*_2_ = *pk*/(*μ* + *k*) is the fraction that survives the early latent period and enters the state of untreated active TB cases;
3. *A*_3_ = *ω*/(*μ* + *ω*) is the fraction that survives the later latent period;
4. *A*_4_ = *r*/(*μ* + *d*_1_ + *r*) is the fraction that survives the state of untreated active TB cases and enters the state of treated active TB cases;
5. *A*_5_ = *γ*/(*μ* + *d*_2_ + *γ* + *ξ*) is the fraction that survives the state of treated active TB cases and enters the state of interrupted treatment;
6. *A*_6_ = (1 − *q*)*η*/(*μ* + *d*_3_ + *η*) is the fraction that survives the state of interrupted treatment and enters the early latent state;
7. *A*_7_ = *qη*/(*μ* + *d*_3_ + *η*) is the fraction that survives the state of interrupted treatment and enters the later latent state;
8. *A*_3_ *A*_5_ *A*_7_ + *A*_1_ *A*_3_ *A*_5_ *A*_6_ + *A*_2_ *A*_5_ *A*_6_ is the fraction that relapses back into the state of untreated active TB cases;
9. 1/((*μ* + *d*_1_ + *r*) − *A*_3_ *A*_5_ *A*_7_ *r* − *A*_1_ *A*_3_ *A*_5_ *A*_6_ *r* − *A*_2_ *A*_5_ *A*_6_ *r*) is the average infectious period of untreated active TB cases;
10. *β*_1_(Λ/*μ*)(*A*_2_ + *A*_1_ *A*_3_)/((*μ* + *d*_1_ + *r*) − *A*_3_ *A*_5_ *A*_7_ *r* − *A*_1_ *A*_3_ *A*_5_ *A*_6_ *r* − *A*_2_ *A*_5_ *A*_6_ *r*) is the average number of the susceptibles being infected by one untreated TB active case during its entire average infectious period;
11. the second term and the third term of *R*_*ti*_ denote the average number of the susceptibles being infected by one treated active TB case and one active TB case who has interrupted treatment during its entire average infectious period, respectively.

Thus, the control reproduction number in this case is the sum of the secondary infections. The paper [13, Theorem  2] implies the following theorem.

**Theorem 1**

If the control reproduction number *R*_*ti*_ < 1, the disease-free equilibrium *P*_0_ is locally asymptotically stable, while if the control reproduction number *R*_*ti*_ > 1, the disease-free equilibrium *P*_0_ is unstable.

One is now in the position to give the existence of the endemic equilibrium of model (2).

**Theorem 2**

If the control reproduction number *R*_*ti*_ > 1, in the TB transmission model (2) there exists exactly one endemic equilibrium *P*_∗_(S*, *E*_1_*, *E*_2_*, I*, T*, D*), where

(9) $$\begin{array}{c} S^{*}=\frac{\Lambda }{\mu R_{ti}},\\ I^{*}=\frac{\mu \left(R_{ti}-1\right)}{\beta_{1}+\left(\beta_{2}r/\left(\mu +d_{2}+\gamma +\xi \right)\right)+\left(\beta_{3}A_{5}r/\left(\mu +d_{3}+\eta \right)\right)},\\ E_{1}^{*}=\frac{A_{8}}{pk+A_{3}\left(1-p\right)k}I^{*},\\ E_{2}^{*}=\frac{\left(1-p\right)k}{\mu +\omega }E_{1}^{*}+\frac{A_{5}q\eta r}{\left(\mu +\omega \right)\left(\mu +d_{3}+\eta \right)}I^{*},\\ T^{*}=\frac{r}{\mu +d_{2}+\gamma +\xi }I^{*},\;\;D^{*}=\frac{A_{5}r}{\mu +d_{3}+\eta }I^{*}. \end{array}$$

**Proof**

It is worth to note that *R*_*ti*_ > 1. Letting the right hand sides of the equations in system (2) to be equal to zero, we get

(10) $$\begin{array}{c} \Lambda =\beta_{1}S^{*}I^{*}+\beta_{2}S^{*}T^{*}+\beta_{3}S^{*}D^{*}+\mu S^{*},\\ \beta_{1}S^{*}I^{*}+\beta_{2}S^{*}T^{*}+\beta_{3}S^{*}D^{*}+\left(1-q\right)\eta D^{*}=\left(\mu +k\right)E_{1}^{*},\\ \left(1-p\right)kE_{1}^{*}+q\eta D^{*}=\left(\mu +\omega \right)E_{2}^{*},\\ pkE_{1}^{*}+\omega E_{2}^{*}=\left(\mu +d_{1}+r\right)I^{*},\\ rI^{*}=\left(\mu +d_{2}+\gamma +\xi \right)T^{*},\\ \gamma T^{*}=\left(\mu +d_{3}+\eta \right)D^{*}. \end{array}$$

The fifth formula of (10) implies

(11) $$\begin{array}{c} T^{*}=\frac{r}{\mu +d_{2}+\gamma +\xi }I^{*}. \end{array}$$

Substituting (11) into the sixth formula of (10) leads to

(12) $$\begin{array}{c} D^{*}=\frac{A_{5}r}{\mu +d_{3}+\eta }I^{*}. \end{array}$$

Equation (12), together with the third formula of (10), yields

(13) $$\begin{array}{c} E_{2}^{*}=\frac{\left(1-p\right)k}{\mu +\omega }E_{1}^{*}+\frac{A_{5}q\eta r}{\left(\mu +\omega \right)\left(\mu +d_{3}+\eta \right)}I^{*}. \end{array}$$

Equation (13) and the fourth formula of (10) yield

(14) $$\begin{array}{c} E_{1}^{*}=\frac{A_{8}}{pk+A_{1}\left(1-p\right)k}I^{*}. \end{array}$$

By substituting (11), (12), and (14) into the second formula of (10), we obtain

(15) $$\begin{array}{c} S^{*}=\frac{\Lambda }{\mu R_{ti}}. \end{array}$$

By using (11), (12), (15), and the first formula of (10), we have

(16) I∗=μ(Rti−1)×(β1+β2rμ+d2+γ+ξ+β3A5rμ+d3+η)−1,

which ends the proof.

## 3. The Stability Analysis

In this section, the stabilities of the equilibria are discussed by constructing the so-called Lyapunov functions [9, 14, 15]. The following theorem states the global stability of the disease-free equilibrium of system (2).

**Theorem 3**

If the control reproduction number *R*_*ti*_ < 1, the disease-free equilibrium *P*_0_ is globally asymptotically stable.

**Proof**

Construct the following Lyapunov function:

(17) $$\begin{array}{c} U_{1}=E_{1}+B_{1}E_{2}+B_{2}I+B_{3}T+B_{4}D, \end{array}$$

where

(18) $$\begin{array}{c} B_{1}=\frac{A_{3}}{A_{2}+A_{1}A_{3}},\;\;B_{2}=\frac{B_{1}}{A_{3}},\\ B_{3}=\frac{\beta_{1}\left(\Lambda /\mu \right)}{\mu +d_{2}+\gamma +\xi }+B_{4}A_{5},\\ B_{4}=\frac{\beta_{3}\left(\Lambda /\mu \right)}{\mu +d_{3}+\eta }+A_{6}+B_{1}A_{7}. \end{array}$$

Differentiating *U*_1_ along with the solutions of the system (2) with respect to time *t* gives

(19) $$\begin{array}{c} {\frac{dU_{1}}{dt}|}_{\left(2\right)}=\frac{dE_{1}}{dt}+B_{1}\frac{dE_{2}}{dt}+B_{2}\frac{dI}{dt}+B_{3}\frac{dT}{dt}+B_{4}\frac{dD}{dt}. \end{array}$$

Substituting the equations of system (2) associated with *E*_1_, *E*_2_, *I*, *T*, and *D* into (19) yields

(20) dU1dt|(2)=β1SI+β2ST+β3SD+(1−q)ηD−(μ+k)E1+B1[(1−p)kE1+qηD−(μ+ω)E2]+B2[pkE1+ωE2−(μ+d1+r)I]+B3[rI−(μ+d2+γ+ξ)T]+B4[γT−(μ+d3+η)D].

Equation (20) can be rewritten as

(21) dU1dt|(2)=[B1(1−p)k+B2pk−(μ+k)]E1+[B2ω−B1(μ+ω)]E2+[β1S+B3r−B2(μ+d1+r)]I+[β2S+B4γ−B3(μ+d2+γ+ξ)]T+[β3S+(1−q)η+B1qη  −B4(μ+d3+η)]D.

By using (18) and the fact that *S* ≤ Λ/*μ*, we have

(22) dU1dt|(2)=[β1S+B3r−B2(μ+d1+r)]I≤[β1Λμ+B3r−B2(μ+d1+r)]I=B2[(μ+d1+r)−A2A5A6r−A3A5A7r  −A1A3A5A6r](Rti−1)I,

with equality only at *P*_0_. For *R*_*ti*_ < 1, this shows (*dU*_1_(*t*))/*dt*|_(2)_ ≤ 0 with equality only if *I* = 0. By LaSalle's invariance principle [16], the limit set of each solution of model (2) is contained in the largest invariant set *I* = 0, which is the singleton {*P*_0_}. This completes the proof.

**Theorem 4**

If the control reproduction number *R*_*ti*_ > 1, the endemic equilibrium *P*_∗_ of the model (2) is globally asymptotically stable.

**Proof**

At the endemic equilibrium *P*_∗_, all the parameters and the components S*, *E*_1_*, *E*_2_*, I*, T*, and D* of the endemic equilibrium *P*_∗_ satisfy the following equations:

(23) $$\begin{array}{c} \Lambda =\beta_{1}S^{*}I^{*}+\beta_{2}S^{*}T^{*}+\beta_{3}S^{*}D^{*}+\mu S^{*},\\ \mu +k=\frac{\beta_{1}S^{*}I^{*}}{E_{1}^{*}}+\frac{\beta_{2}S^{*}T^{*}}{E_{1}^{*}}+\frac{\beta_{3}S^{*}D^{*}}{E_{1}^{*}}+\frac{\left(1-q\right)\eta D^{*}}{E_{1}^{*}},\\ \mu +\omega =\frac{\left(1-p\right)kE_{1}^{*}}{E_{2}^{*}}+\frac{q\eta D^{*}}{E_{2}^{*}},\\ \mu +d_{1}+r=\frac{pkE_{1}^{*}}{I^{*}}+\frac{\omega E_{2}^{*}}{I^{*}},\\ \mu +d_{2}+\gamma +\xi =\frac{rI^{*}}{T^{*}},\\ \mu +d_{3}+\eta =\frac{\gamma T^{*}}{D^{*}}. \end{array}$$

Let the Lyapunov function be as follows:

(24) U2=S−S∗ln⁡S+E1−E1∗ln⁡E1+C1(E2−E2∗ln⁡E2)+C2(I−I∗ln⁡I)+C3(T−T∗ln⁡T)+C4(D−D∗ln⁡D),

where

(25) $$\begin{array}{c} C_{1}=A_{3}C_{2},\\ C_{2}=\frac{\beta_{1}S^{*}I^{*}+\beta_{2}S^{*}T^{*}+\beta_{3}S^{*}D^{*}+\left(1-q\right)\eta D^{*}}{\left[pk+A_{3}\left(1-p\right)k\right]E_{1}^{*}},\\ C_{3}=\frac{\beta_{2}S^{*}T^{*}+C_{4}\gamma T^{*}}{rI^{*}},\\ C_{4}=\frac{\beta_{3}S^{*}D^{*}+\left(1-q\right)\eta D^{*}+C_{1}q\eta D^{*}}{\gamma T^{*}}. \end{array}$$

Differentiating *U*_2_ along the solutions of system (2) with respect to time *t* gives

(26) dU2dt|(2)=(1−S∗S)dSdt+(1−E1∗E1)dE1dt+C1(1−E2∗E2)dE2dt+C2(1−I∗I)dIdt+C3(1−T∗T)dTdt+C4(1−D∗D)dDdt.

Substituting system (2) into (26) yields

(27) dU2dt|(2)=(1−S∗S)[Λ−β1SI−β2ST−β3SD−μS]+(1−E1∗E1)[β1SI+β2ST+β3SD        +(1−q)ηD−(μ+k)E1]+C1(1−E2∗E2)[(1−p)kE1+qηD          −(μ+ω)E2]+C2(1−I∗I)[pkE1+ωE2−(μ+d1+r)I]+C3(1−T∗T)[rI−(μ+d2+γ+ξ)T]+C4(1−D∗D)[γT−(μ+d3+η)D].

Using (23), we have

(28) dU2dt|(2)=(1−S∗S)[β1S∗I∗+β2S∗T∗+β3S∗D∗+μS∗ −β1SI−β2ST−β3SD−μS]+(1−E1∗E1)[β1SI+β2ST+β3SD+(1−q)ηD −β1S∗I∗E1∗E1−β2S∗T∗E1∗E1 −β3S∗D∗E1∗E1−(1−q)ηD∗E1∗E1]+C1(1−E2∗E2)[(1−p)kE1+qηD   −(1−p)kE1∗E2∗E2−qηD∗E2∗E2]+C2(1−I∗I)[pkE1+ωE2−pkE1∗I∗I−ωE2∗I∗I]+C3(1−T∗T)[rI−rI∗T∗T]+C4(1−D∗D)[γT−γT∗D∗D].

The above equation can be rearranged as

(29) dU2dt|(2)=−μ(S∗−S)2S+β1S∗I∗(1−S∗S)(1−SIS∗I∗)+β2S∗T∗(1−S∗S)(1−STS∗T∗)+β3S∗D∗(1−S∗S)(1−SDS∗D∗)+β1S∗I∗(1−E1∗E1)(SIS∗I∗−E1E1∗)+β2S∗T∗(1−E1∗E1)(STS∗T∗−E1E1∗)+β3S∗D∗(1−E1∗E1)(SDS∗D∗−E1E1∗)+(1−q)ηD∗(1−E1∗E1)(DD∗−E1E1∗)+C1(1−p)kE1∗(1−E2∗E2)(E1E1∗−E2E2∗)+C1qηD∗(1−E2∗E2)(DD∗−E2E2∗)+C2pkE1∗(1−I∗I)(E1E1∗−II∗)+C2ωE2∗(1−I∗I)(E2E2∗−II∗)+C3rI∗(1−T∗T)(II∗−TT∗)+C4γT∗(1−D∗D)(TT∗−DD∗).

Let *x* = *S*/S*, *y* = *E*_1_/*E*_1_*, *z* = *E*_2_/*E*_2_*, *u* = *I*/I*, *v* = *T*/T*, and *w* = *D*/D*, and we get

(30) dU2dt|(2)=−μ(S∗−S)2S+β1S∗I∗(1−1x)(1−xu)+β2S∗T∗(1−1x)(1−xv)+β3S∗D∗(1−1x)(1−xw)+β1S∗I∗(1−1y)(xu−y)+β2S∗T∗(1−1y)(xv−y)+β3S∗D∗(1−1y)(xw−y)+(1−q)ηD∗(1−1y)(w−y)+C1(1−p)kE1∗(1−1z)(y−z)+C1qηD∗(1−1z)(w−z)+C2pkE1∗(1−1u)(y−u)+C2ωE2∗(1−1u)(z−u)+C3rI∗(1−1v)(u−v)+C4γT∗(1−1w)(v−w).

That is, to say, we obtain

(31) dU2dt|(2)=−μ(S∗−S)2S+β1S∗I∗(1−xu−1x+u)+β2S∗T∗(1−xv−1x+v)+β3S∗D∗(1−xw−1x+w)+β1S∗I∗(xu−y−xuy+1)+β2S∗T∗(xv−y−xvy+1)+β3S∗D∗(xw−y−xwy+1)+(1−q)ηD∗(w−y−wy+1)+C1(1−p)kE1∗(y−z−yz+1)+C1qηD∗(w−z−wz+1)+C2pkE1∗(y−u−yu+1)+C2ωE2∗(z−u−zu+1)+C3rI∗(u−v−uv+1)+C4γT∗(v−w−vw+1).

Applying the expressions in (25) yields

(32) dU2dt|(2)=−μ(S∗−S)2S+E1∗[2β1S∗I∗E1∗+2β2S∗T∗E1∗+2β3S∗D∗E1∗+(1−q)ηD∗E1∗+C1(1−p)k+C1qηD∗E1∗+C2pk+C2ωE2∗E1∗+C3rI∗E1∗+C4γT∗E1∗]−E1∗[β1S∗I∗E1∗(1x+xuy)+β2S∗T∗E1∗(1x+xvy)    +β3S∗D∗E1∗(1x+xwy)+(1−q)ηD∗E1∗wy    +C1(1−p)kyz+C1qηD∗E1∗wz+C2pkyu    +C2ωE2∗E1∗zu+C3rI∗E1∗uv+C4γI∗E1∗vw].

Applying (9) and (25), it is easy to see that

(33) β1S∗I∗E1∗=β1S∗pk+A3(1−p)kA8=β1S∗pkA8+β1S∗A3(1−p)kA8,β2S∗T∗E1∗=β2S∗pkr(μ+d2+γ+ξ)A8+β2S∗A3(1−p)kr(μ+d2+γ+ξ)A8,β3S∗D∗E1∗=β3S∗A5pkr(μ+d3+η)A8+β3S∗A3A5(1−p)kr(μ+d3+η)A8,(1−q)ηD∗E1∗=A5pk(1−q)ηr(μ+d3+η)A8+A3A5(1−p)k(1−q)ηr(μ+d3+η)A8,C1(1−p)k=A3C2(1−p)k=β1S∗A3(1−p)kA8+β2S∗A3(1−p)kr(μ+d3+η)A8+β3S∗A3A5(1−p)kr(μ+d3+η)A8+A3A5(1−p)k(1−q)ηr(μ+d3+η)A8,C2pk=β1S∗pkA8+β2S∗pkr(μ+d3+η)A8+β3S∗A5pkr(μ+d3+η)A8+A5(1−q)ηpkr(μ+d3+η)A8,C2ωE2∗E1∗=C2[A3(1−p)k+A3A5A7r(pk+A3(1−p)k)A8]=β1S∗A3(1−p)kA8+β2S∗A3(1−p)kr(μ+d3+η)A8+β3S∗A3A5(1−p)kr(μ+d3+η)A8+A3A5(1−p)k(1−q)ηr(μ+d3+η)A8+C1qηD∗E1∗,C3rI∗E1∗=β2S∗pkr(μ+d2+γ+ξ)A8+β2S∗A3(1−p)kr(μ+d2+γ+ξ)A8+β3S∗A5pkr(μ+d3+η)A8+β3S∗A3A5(1−p)kr(μ+d3+η)A8+A5pk(1−q)ηr(μ+d3+η)A8+A3A5(1−p)k(1−q)ηr(μ+d3+η)A8+C1qηD∗E1∗,C4γIE1∗=β3S∗A5pkr(μ+d3+η)A8+β3S∗A3A5(1−p)kr(μ+d3+η)A8+A5pk(1−q)ηr(μ+d3+η)A8+A3A5(1−p)k(1−q)ηr(μ+d3+η)A8+C1qηD∗E1∗.

By using (33), (32) can be rewritten as follows:

(34) dU2dt|(2)=−μ(S∗−S)2S+E1∗[3β1S∗pkA8+4β1S∗A3(1−p)kA8 +4β2S∗pkγ(μ+d2+γ+ξ)A8 +5β2S∗A3(1−p)kr(μ+d2+γ+ξ)A8 +5β3S∗A5pkγ(μ+d3+η)A8 +6β3S∗A3A5(1−p)kr(μ+d3+η)A8 +4A5pk(1−q)ηr(μ+d3+η)A8 +5A3A5(1−p)k(1−q)ηr(μ+d3+η)A8 +4C1qηD∗E1∗]−E1∗[β1S∗pkA8(1x+xuy+yu)+β1S∗A3(1−p)kA8(1x+xuy+yz+zu)+β2S∗pkγ(μ+d2+γ+ξ)A8(1x+xvy+yu+uv)+β2S∗A3(1−p)kr(μ+d2+γ+ξ)A8×(1x+xvy+yz+zu+uv)+β3S∗A5pkγ(μ+d3+η)A8×(1x+xwy+yu+uv+vw)+β3S∗A3A5(1−p)kr(μ+d3+η)A8×(1x+xwy+yz+zu+uv+vw)+A5pk(1−q)ηr(μ+d3+η)A8(wy+vw+yu+uv)+A3A5(1−p)k(1−q)ηr(μ+d3+η)A8×(wy+yz+vw+zu+uv)+C1qηD∗E1∗(wz+vw+zu+uv)].

By the inequality of the arithmetic mean-geometric mean, we get

(35) $$\begin{array}{c} {\frac{dU_{2}}{dt}|}_{\left(2\right)}\leq -\mu \frac{{\left(S^{*}-S\right)}^{2}}{S}\leq 0, \end{array}$$

with equality if and only if *x* = 1 and *y* = *z* = *u* = *v* = *w*.

Combining those inequalities, we have that (*dU*_2_(*t*))/*dt*|_(2)_ ≤ 0 with equality only if *S* = S*, *E*_1_ = *E*_1_*, *E*_2_ = *E*_2_*, *I* = I*, *T* = T*, and *D* = D*. Therefore, an application of the LaSalle invariance principle [5] yields that the endemic equilibrium {*P*_∗_} is globally asymptotically stable in *Ω*.

**Figure 2:**
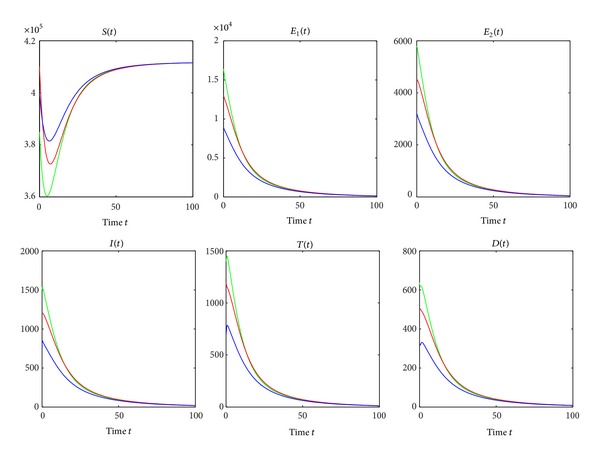
The global asymptotic stability of the disease-free equilibrium *P*_0_ when *R*_*ti*_ = 0.9338. The green line, the red line, and the blue line correspond to the initial values (3.85 × 10^5^, 1.64 × 10^4^,5.8 × 10^3^, 1.5 × 10^3^, 1.4 × 10^3^, 640), (4.1 × 10^5^, 1.3 × 10^4^, 4.5 × 10^3^, 1.2 × 10^3^, 1.2 × 10^3^, 500), and (4 × 10^5^, 9 × 10^3^, 3.2 × 10^3^, 750, 700, 330), respectively. *β*_2_ is fixed as 0.00000035. Other parameter values can be seen in Table 1.

**Figure 3:**
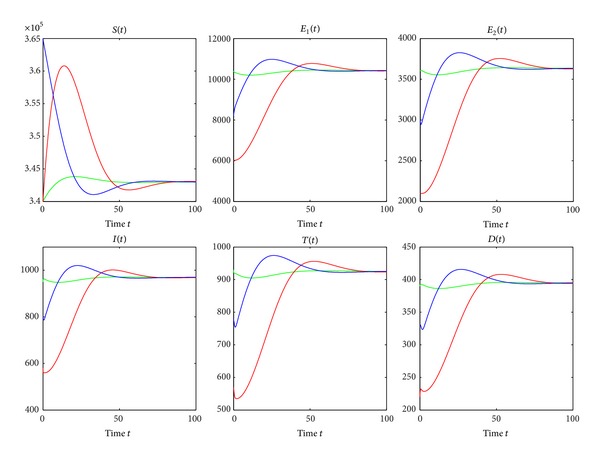
The global asymptotic stability of the endemic equilibrium *P*_∗_ when *R*_*ti*_ = 1.2006. The green line, the red line, and the blue line correspond to the initial values (3.4 × 10^5^, 1.04 × 10^4^, 3.6 × 10^3^, 950, 930, 390), (3.4 × 10^5^, 6 × 10^3^, 2.1 × 10^3^, 580, 570, 220), and (3.65 × 10^5^, 8.2 × 10^3^, 3 × 10^3^, 800, 780, 330), respectively. We chose *β*_2_ = 0.00000045. Other parameter values are in Table 1.

From Theorems 3 and 4, we see that *R*_*ti*_ is a sharp threshold parameter to determine whether or not the TB is epidemic in the population. Furthermore, Figure 2 verifies the theoretical analysis that the disease-free equilibrium *P*_0_ is globally asymptotically stable when *R*_*ti*_ = 0.9338 < 1. Numerical simulation illustrates that there exists a global asymptotical stable endemic equilibrium *P*_∗_ when *R*_*ti*_ = 1.2006 > 1 (see Figure 3), where year is used as the unit of time.

## 4. Effects of Treatment and Treatment Interruptions

The first line antituberculosis drugs are taken daily for the first two months of intensive phase and rifampicin and isoniazid are taken daily for the later four months of continuation phase in order to treat active TB cases. However, the treatment interruptions frequently occur during the period of treatment due to a great number of reasons [4], which may be one of the main factors to cause drug-resistant TB cases. We now discuss the effects of treatment of active TB cases and treatment interruptions on the development of TB. It is assumed that if the treatment rate of active TB cases *r* = 0, the rate of treatment interruptions *γ* must be zero because the treatment interruptions occur during the period of treatment of active TB cases.

If there is no treatment of active TB cases, in fact, and there are no treatment interruptions either, we have *r* = 0 and *γ* = 0. The system (2) becomes

(36) dSdt=Λ−β1SI−μS,dE1dt=β1SI−(μ+k)E1,dE2dt=(1−p)kE1−(μ+ω)E2,dIdt=pkE1+ωE2−(μ+d1)I.

Hence, the basic reproduction number of system (36) is given by

(37) $$\begin{array}{c} \underset{\left(r,\gamma \right)\to \left(0,0\right)}{\lim}R_{ti}=\frac{\beta_{1}\left(\Lambda /\mu \right)\left(A_{2}+A_{1}A_{3}\right)}{\mu +d_{1}}=:R_{0}. \end{array}$$

*R*_0_ can be interpreted as the number of secondary infections caused by one active TB case introduced into the population which is made up of susceptible individuals during its entire infectious period.

If there are no treatment interruptions during the period of treatment of active TB cases, in other words, *γ* = 0, then the system (2) becomes

(38) dSdt=Λ−β1SI−β2ST−μS,dE1dt=β1SI+β2ST−(μ+k)E1,dE2dt=(1−p)kE1−(μ+ω)E2,dIdt=pkE1+ωE2−(μ+d1+r)I,dTdt=rI−(μ+d2+ξ)T,

which implies that

(39) lim⁡γ→0Rti=β1(Λ/μ)(A2+A1A3)μ+d1+r+β2(Λ/μ)(A2+A1A3)A4μ+d2+ξ=:Rt,

where *R*_*t*_ is the treatment induced reproduction number for model (38). *R*_*t*_ is the sum of the numbers of secondary infections caused by one untreated and one treated active TB cases introduced into the population during its entire infectious period. Furthermore,

(40) $$\begin{array}{c} \underset{r\to 0}{\lim}R_{t}=R_{0}. \end{array}$$

Differentiating partially *R*_*t*_ with respect to *r*, we obtain

(41) $$\begin{array}{c} \frac{\partial R_{t}}{\partial r}=\frac{-\left(\Lambda /\mu \right)\left(A_{2}+A_{1}A_{3}\right)\beta_{2}\Delta_{1}}{{\left(\mu +d_{1}+r\right)}^{2}}, \end{array}$$

where

(42) $$\begin{array}{c} \Delta_{1}=\frac{\beta_{1}}{\beta_{2}}-\frac{\mu +d_{1}}{\mu +d_{2}+\xi }. \end{array}$$

It is quite reasonable to require that the treatment of active TB cases is effective, which implies *R*_0_ > *R*_*t*_. In other words, ∂*R*_*t*_/∂*r* < 0 and Δ_1_ > 0 hold. In fact, when Δ_1_ > 0 is satisfied, treatment of active TB cases slows down the TB epidemic if there are no treatment interruptions during the period of treatment of active TB cases.

For the sake of simplicity, let

(43) Δ2=β1β2(1−A2A5A6−A3A5A7−A1A3A5A6)−μ+d1μ+d2+γ+ξ−β3A5(μ+d1)β2(μ+d3+η),

(44) Δ3=(β3(μ+d2+ξ)β2(μ+d3+η)−1)r+(β1(μ+d2+ξ)β2+r)×(A3A4A7+A2A4A6+A1A3A4A6).

Differentiating partially *R*_*ti*_ with respect to *r*, we get

(45) $$\begin{array}{c} \frac{\partial R_{ti}}{\partial r}=\frac{-\left(\Lambda /\mu \right)\left(A_{2}+A_{1}A_{3}\right)\beta_{2}\Delta_{2}}{{\left(A_{9}\right)}^{2}}. \end{array}$$

From the epidemiological point of view, it is assumed that Δ_2_ > 0, which implies that the treatment of active TB cases reduces the TB epidemic. It is natural epidemiologically to assume that both Δ_1_ > 0 and Δ_2_ > 0 in the present paper. Differentiating partially *R*_*ti*_ with respect to *γ* gives

(46) $$\begin{array}{c} \frac{\partial R_{ti}}{\partial \gamma }=\frac{\left(\Lambda /\mu \right)\left(A_{2}+A_{1}A_{3}\right)\left(\mu +d_{1}+r\right)\beta_{2}\Delta_{3}}{{\left(A_{9}\right)}^{2}{\left(\mu +d_{2}+\gamma +\xi \right)}^{2}} \end{array}$$

- when Δ_3_ > 0, *R*_*ti*_ > *R*_*t*_, and ∂*R*_*ti*_/∂*γ* > 0;
- when Δ_3_ < 0, *R*_*ti*_ < *R*_*t*_, and ∂*R*_*ti*_/∂*γ* < 0;
- and when Δ_3_ = 0, *R*_*ti*_ ≡ *R*_*t*_, and ∂*R*_*ti*_/∂*γ* = 0.

That is to say that treatment interruptions may reduce or accelerate the TB epidemic or may have no effect on the TB epidemic.

From (46), we pay attention to the following scenarios.

**Case 1 (Δ_3_ > 0)**

In this case, treatment interruptions have a negative effect on the development of TB because of ∂*R*_*ti*_/∂*γ* > 0. *R*_*ti*_ ≥ *R*_0_ implies that treatment of active TB cases is not enough to compensate for the treatment interruptions. Therefore, according to the practical situation, it is sufficient to consider the case of *R*_*ti*_ < *R*_0_, and then we get *R*_*t*_ < *R*_*ti*_ < *R*_0_. If *R*_0_ < 1, TB will be eradicated from the population and treatment of active TB cases is not necessary.

If *R*_*ti*_ < 1 < *R*_0_ is valid, it is necessary to take measures to prevent the spread of TB, for instance, treatment of active TB cases. We have to determine the necessary conditions for slowing down the TB epidemic. If active TB cases are treated and there are treatment interruptions, setting *R*_*ti*_ = 1, we obtain the critical values of treatment rate of active TB cases and the rate of treatment interruptions denoted by *r*_1_^*c*^ and *γ*_1_^*c*^, respectively. Moreover, *r*_1_^*c*^ and *γ*_1_^*c*^ satisfy the following equation:

(47) Λμ(A2+A1A3)[β1(μ+d2+γ1c+ξ)   +β2r1c+β3μ+d3+ηr1cγ1c] ×((μ+d1+r1c)(μ+d2+γ1c+ξ)   −(A2A6+A3A7+A1A3A6)r1cγ1c)−1=1.

Clearly, when one of the conditions

(c1) *r* > *r*_1_^*c*^ and *γ* = *γ*_1_^*c*^;
(c2) *r* = *r*_1_^*c*^ and *γ* < *γ*_1_^*c*^;
(c3) *r* > *r*_1_^*c*^ and *γ* < *γ*_1_^*c*^

is fulfilled, TB will not develop an epidemic and will die out from the population.

If *R*_*t*_ < 1 < *R*_*ti*_, it should be noted that reducing the existing rate of treatment interruptions can slow down the TB epidemic when the treatment rate of active TB cases remains the same. Setting *R*_*ti*_ = 1, we obtain

(48) γ2c=(Λμ(A2+A1A3)[β1(μ+d2+ξ)+β2r] −(μ+d1+r)(μ+d2+ξ))×(μ+d1+(1−A2A6−A3A7−A1A3A6)r   −Λμ(A2+A1A3)(β1+β3rμ+d3+η))−1.

When *γ* < *γ*_2_^*c*^, TB will be controlled and does not develop the epidemic; otherwise reducing the rate of treatment interruptions results in the reduction of TB epidemic but not enough to control TB.

If *R*_*t*_ > 1, the existing treatment of active TB cases will cut down the spread of TB but is not enough to control TB. The possible best way is to improve the existing treatment rate of active TB cases or more other steps should be taken in order to control TB.

**Case 2 (Δ_3_ < 0)**

In this case, both treatment of active TB cases and treatment interruptions slow down the development of TB epidemic. Furthermore, we have *R*_*ti*_ < *R*_*t*_ < *R*_0_. If *R*_0_ < 1, TB can not develop into epidemic and treatment of active TB cases is not necessary either, but treatment of active TB cases and treatment interruptions may accelerate the extinction of TB.

If *R*_*t*_ < 1 < *R*_0_, it is necessary to determine the condition for slowing down the spread of TB. Let *R*_*t*_ = 1 and solve the critical treatment rate of active TB cases denoted by *r*_2_^*c*^ if there are no treatment interruptions, we get

(49) r2c=β1(Λ/μ)(A2+A1A3)(μ+d2+ξ)(μ+d2+ξ)−β2(Λ/μ)(A2+A1A3)−(μ+d1)(μ+d2+ξ)(μ+d2+ξ)−β2(Λ/μ)(A2+A1A3).

When *r* > *r*_2_^*c*^, TB will be eradicated due to treatment of active TB cases only. If *r* < *r*_2_^*c*^, there is a reduction in the TB epidemic but it is not enough to eradicate the TB epidemic.

If *R*_*ti*_ < 1 < *R*_*t*_, we need to determine the condition for slowing down the development of TB epidemic. It follows from letting *R*_*ti*_ = 1 that the critical value of rate of treatment interruptions denoted by *γ*_3_^*c*^ is obtained, which is exactly the same as *γ*_2_^*c*^. When *γ* > *γ*_3_^*c*^, TB will be controlled and die out from the population eventually. On the contrary, if *γ* < *γ*_3_^*c*^, treatment interruptions slow down the development of TB epidemic but TB will not be eradicated through the strategy of treatment interruptions only.

If *R*_*ti*_ > 1, then the existing treatment of active TB cases and the existing treatment interruptions can not control the epidemic and other intervention strategies should be introduced.

**Case 3 (Δ_3_ = 0)**

In this case, treatment interruptions have no effect on the development of TB epidemic due to ∂*R*_*ti*_/∂*γ* = 0 or *R*_*ti*_ ≡ *R*_*t*_. If *R*_0_ < 1, TB will eventually disappear from the population and treatment of active TB cases is not necessary. On the contrary, if *R*_0_ > 1, we determine the critical treatment rate. Let *R*_*t*_ = 1; the critical treatment rate denoted by *r*_3_^*c*^ is obtained, which is the same as *r*_2_^*c*^. When *r* > *r*_3_^*c*^, TB will be eradicated through treatment of active TB cases. But if *r* < *r*_3_^*c*^, treatment of active TB cases results in the reduction of the TB epidemic but not enough to control TB.

**Figure 4:**
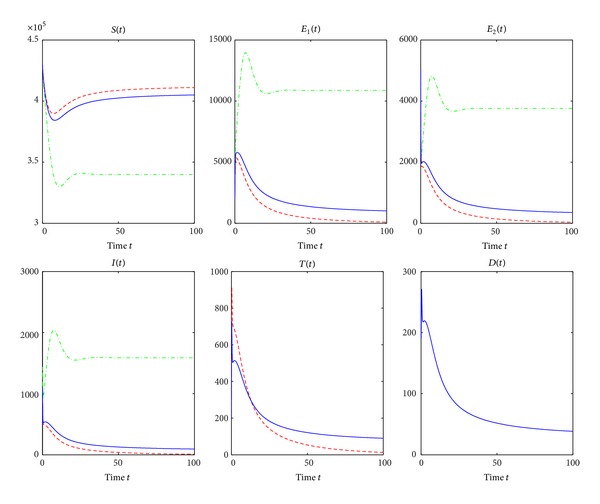
Simulation results showing the effects of treatment and treatment interruptions on all compartments. The green dash-dotted line, the red dashed line, and the blue solid line correspond to no treatment of active TB cases, treatment of active TB cases only, and treatment of active TB cases and treatment interruptions, respectively. *β*_2_ = 0.00000038, and other parameter values are seen in Table 1. Δ_3_ is calculated as 0.1513 > 0.

**Figure 5:**
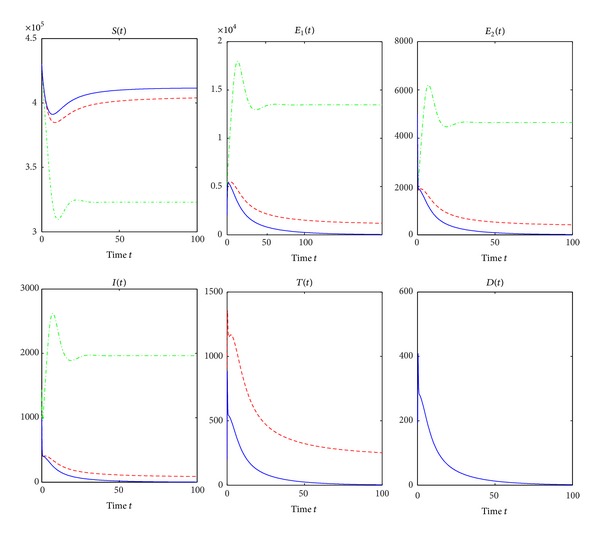
Simulation results showing the effects of treatment and treatment interruptions. The green dash-dotted line, the red dashed line, and the blue solid line correspond to no treatment of active TB cases, treatment of active TB cases only, and treatment of active TB cases and treatment interruptions, respectively. *d*_3_ = 0.35, *β*_2_ = 0.0000004, *γ* = 0.2, *r* = 0.5, and *ξ* = 0.06. Other parameter values can be found in Table 1. And Δ_3_ = −0.1275 < 0.

We are now doing numerical simulations for models (2), (36), and (38). Many parameter values used for the numerical simulations are listed in Table 1. Most of the parameter values are from [17] and others are estimated from the relatively reasonable point of view. For the simulation of the result in Figures 4 and 5, the initial conditions assumed that *S*(0) = 430,000, *E*_1_(0) = 2,000, *E*_2_(0) = 5,000, *I*(0) = 500, *T*(0) = 200, and *D*(0) = 250.

Figure 4 is a graphical representation demonstrating the trends of all classes where there is no treatment and when treatment of active TB cases is used. *β*_2_ is chosen as 0.00000038 and other parameter values are seen in Table 1. The green dash-dotted line, the red dashed line, and the blue solid line correspond to no treatment, treatment of active TB cases but no treatment interruptions, and treatment of active TB cases and treatment interruptions, respectively. It is easy to calculate that Δ_1_ = 2.6 > 0, Δ_2_ = 2.1911 > 0, Δ_3_ = 0.1513 > 0, *R*_0_ = 1.2112, *R*_*t*_ = 0.9791, and *R*_*ti*_ = 1.0138, which implies that *R*_*t*_ < 1 < *R*_*ti*_ < *R*_0_. The numbers of susceptible people decrease as susceptible people are infected by active TB cases. However, they stop falling and soon reach points where they remain constants as shown in the first panel of Figure 4. However, the susceptibles, in the absence of treatment of active TB cases, are reduced to the lowest level because more susceptibles are infected by active TB cases. Furthermore, in the presence of treatment of active TB cases but no treatment interruptions, the number of susceptible population reaches the biggest stable state as more active TB cases are treated. In Figure 4, in the absence of treatment, the numbers of other three classes increase rapidly as susceptible people are infected, then reach their maximums, then gradually fall, and then reach points where they keep constant. In the presence of treatment of active TB cases and treatment interruptions as shown in Figure 4, the numbers of other five classes fall off and are reduced to lower levels. If there is treatment of active TB cases but no treatment interruptions, the numbers of the other four classes gradually diminish and ultimately reach the stable states zero as shown in Figure 4.

Figure 5 is also a graphical representation showing the trends of all classes where there is no intervention and when treatment of active TB cases and treatment interruptions are applied. The green dash-dotted line, the red dashed line, and the blue solid line correspond to no treatment of active TB cases, treatment of active TB cases only, and treatment of active TB cases and treatment interruptions, respectively. *d*_3_ = 0.35, *β*_2_ = 0.0000004, *γ* = 0.2, *r* = 0.5, and *ξ* = 0.06. Other parameter values can be seen in Table 1. Simple calculation gives Δ_1_ = 2.0492 > 0, Δ_2_ = 2.4254 > 0, and Δ_3_ = −0.1275 < 0. *R*_0_ = 1.2749, *R*_*t*_ = 1.0173, and *R*_*ti*_ = 0.9661. Therefore, *R*_*ti*_ < 1 < *R*_*t*_ < *R*_0_. From Figures 4 and 5, in the absence of treatment of active TB cases, all classes have very similar trends whether or not Δ_3_ > 0. In the presence of treatment, the numbers of susceptible population decrease as susceptible population is infected by active TB cases and then increase to points where they remain constant whether or not there are treatment interruptions in the first panel of Figure 5. However, in the presence of treatment of active TB cases and treatment interruptions, the susceptible population is to reach the highest stable state as shown in the first panel of Figure 5. In Figure 5, in the treatment of active TB cases only, all other populations except for susceptible populations gradually decline and reach the lower stable states, respectively. However, in the presence of treatment of active TB cases and treatment interruptions, the numbers of all classes except for the susceptibles decrease to zero quickly as shown in Figure 5 as there are very few persons who survive the infectious period of active TB cases who have interrupted treatment.

**Figure 6:**
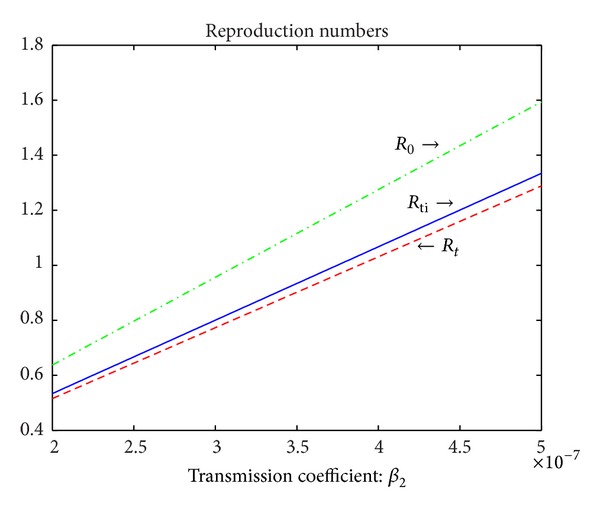
Trends of the reproduction numbers *R*_0_, *R*_*t*_, and *R*_*ti*_. The green dash-dotted line, the red dashed line, and the blue solid line correspond to the reproduction numbers *R*_0_, *R*_*t*_, and *R*_*ti*_, respectively. *β*_2_ is varied from 2 × 10^−7^ to 5 × 10^−7^. Other parameter values can be seen in Table 1. Therefore, Δ_3_ = 0.1513 > 0.

**Figure 7:**
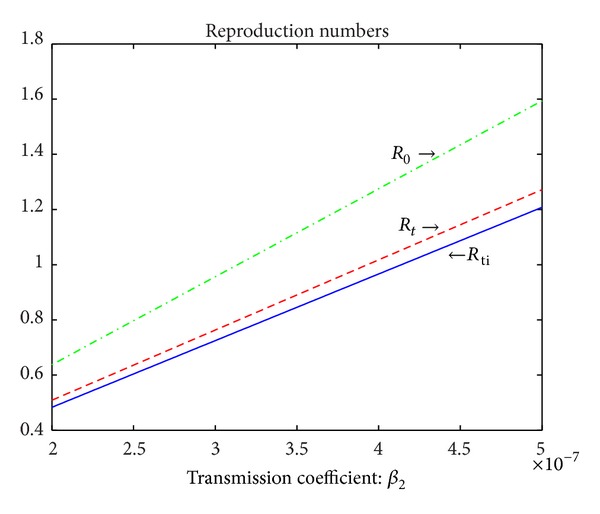
The relationships between the reproduction numbers *R*_0_, *R*_*t*_, and *R*_*ti*_ and the transmission coefficient *β*_2_. The green dash-dotted line, the red dashed line, and the blue solid line correspond to the reproduction numbers *R*_0_, *R*_*t*_, and *R*_*ti*_, respectively. *β*_2_ is varied from 2 × 10^−7^ to 5 × 10^−7^. *d*_3_ = 0.35, *γ* = 0.2, *r* = 0.5, and *ξ* = 0.06. Other parameter values are given in Table 1. And hence Δ_3_ = −0.1275 < 0.

Figures 6 and 7 show the trends of all reproduction numbers as transmission coefficient *β*_2_ is varied. The green dash-dotted line, the red dashed line, and the blue solid line represent the reproduction numbers *R*_0_, *R*_*t*_, and *R*_*ti*_, respectively. In Figure 6, other parameter values are given in Table 1. In Figure 7, *d*_3_ = 0.35, *γ* = 0.2, *r* = 0.5, *ξ* = 0.06, and other parameter values can be seen in Table 1. In Figure 6, Δ_3_ = 0.1513 > 0, which implies treatment interruptions have nagetive effect on the control and prevention of TB. However, in Figure 7, Δ_3_ = −0.1275 < 0 illustrating that treatment interruptions help to control TB as the treatment rate of active TB cases, the rate of treatment interruptions, and the induced death rate in class *D* are increased and the recovery rate of the treated active TB cases is decreased, which is equivalent to the fact that more active TB cases die due to TB. Comparing the results in Figures 6 and 7, it is concluded that both improving the treatment rate of active TB cases and reducing the rate of treatment interruptions may be the effective approaches to control TB epidemic when Δ_3_ is greater than zero. However, if Δ_3_ is less than zero, the possible better way to defeat the TB epidemic is to increase the treatment rate of active TB cases or the rate of treatment interruptions.

## 5. Conclusion

A mathematical TB model with treatment interruptions and two latent periods has been developed in the present paper. The global stability of the model can be completely decided by the threshold parameter *R*_*ti*_. It is shown that the disease-free equilibrium is globally asymptotically stable and TB will die out in the population if the control reproduction number *R*_*ti*_ is below one; and the unique endemic equilibrium is globally asymptotically stable and TB will persist in the population if the control reproduction number *R*_*ti*_ is greater than one. The obtained numerical results verify that treatment of active TB cases always slows down the development of TB epidemic and helps to control the spread of TB. However, if the disease induced death rate *d*_3_ of class *D* is lower, the control reproduction number *R*_*ti*_ increases as the rate of treatment interruptions *γ* increases and treatment interruptions are disadvantageous to control TB epidemic, while if the disease induced death rate *d*_3_ of class *D* is higher, the control reproduction number *R*_*ti*_ decreases as the rate of treatment interruptions *γ* increases and treatment interruptions may be able to slow down the TB epidemic.

## Figures and Tables

**Table 1:** Model parameters and their interpretations.

Definition	Symbol	Estimate
Recruitment rate	Λ	10,000/1.7
Natural death rate	μ	1/70
TB induced death rate in class *I*	*d*_1_	0.5
TB induced death rate in class *T*	*d*_2_	0.1
TB induced death rate in class *D*	*d*_3_	0.2
Transmission coefficient from class *I* to *S*	β_1_	5β_2_
Transmission coefficient from class *T* to *S*	β_2_	Variable
Transmission coefficient from class *D* to *S*	β_3_	1.5β_2_
Reactivation rate of the early latent persons	*k*	0.08
Reactivation rate of the later long-term latent persons	ω	0.2
Self-cured rate of the persons in class *D*	η	0.02
Treatment rate of untreated active TB cases in class *I*	*r*	0.3
Recovery rate of the treated active TB cases	ξ	0.1
Rate of treatment interruptions	γ	0.1
Fraction of the early latent persons who progress TB fast	*p*	0.075
Fraction of the self-cured persons in class *D* who enter class *E*_2_	*q*	0.9
